# Composite risk of cardiovascular disease comorbidity in people living with diabetes in Africa

**DOI:** 10.1097/XCE.0000000000000347

**Published:** 2025-10-16

**Authors:** Regina Idu Ejemot-Nwadiaro, Divine-Favour Chichenim Ofili, Stephen Chukwuma Ogbodo, Henshaw Okoroiwu, Ugochinyere Vivian Ukah

**Affiliations:** a Department of Public Health, School of Allied Health Sciences; b Directorate of Research, Innovation, Consultancy, and Extension, Kampala International University, Ishaka, Uganda; c Department of Public Health, Faculty of Allied Medical Sciences, University of Calabar, Calabar, Nigeria; d Department of Epidemiology, Biostatistics and Occupational Health, Faculty of Medicine and Health Sciences, McGill University; e Lady Davis Institute for Medical Research, Jewish General Hospital, Montreal, Quebec, Canada; f Department of Medical Laboratory Sciences; g International Institute for Oncology and Cancer Research, David Umahi Federal University of Health Sciences, Uburu, Ebonyi State, Nigeria; h Department of Medicine, Faculty of Medicine and Health Sciences, McGill University, Montreal, Quebec, Canada

**Keywords:** Africa, cardiovascular diseases, cardiovascular diseases risk assessment, diabetes, diabetes mellitus–CVD comorbidity, risk stratification, STEPS-CARDIO index

## Abstract

**Aim:**

Despite the high likelihood of comorbidity from shared risk factors, the cardiovascular disease (CVD) risk, based on the combined presence of key risk factors, in people with diabetes mellitus is poorly understood. Thus, this study quantifies the composite CVD risk and its association with sociodemographic factors in people living with diabetes mellitus in Africa.

**Methods:**

Using the WHO STEPwise approach to noncommunicable diseases risk factor Surveillance (STEPS) data and generalized linear mixed models, we analyzed CVD risk in 4738 nonpregnant adults with diabetes in Africa. The composite CVD risk was measured with the STEPS-CARDIO index, which includes smoking, overweight/obesity, hypertension, physical activity, and diet.

**Results:**

The mean STEPS-CARDIO score ranged from 2 to 3.22, signifying that, on average, people with diabetes in the included countries had a medium risk of developing CVD. Higher CVD risk was observed with increasing age [odds ratio (OR): 1.03, 95% confidence interval (CI): 1.02–1.04], and among women compared with men (OR: 1.20, 95% CI: 1.05–1.38). Other factors – education, marital status, occupation, and household size – were also generally associated with elevated CVD risk, but being a student (compared to being employed) was associated with lower risk (OR: 0.51, 95% CI: 0.31–0.86). Additional analyses suggested that this protective association was partly age-driven, as most students were younger than 30 years.

**Conclusion:**

Findings highlight gendered and social differences in CVD risk among people with diabetes mellitus in Africa, underscoring the need for tailored preventive interventions.

## Introduction

The global burden of diabetes mellitus is projected to exceed 852 million cases by 2050, with the steepest increases expected in low- and middle-income countries (LMICs), where underdiagnosis is widespread [1]. In Africa, more than 70% of adults with diabetes remain undiagnosed [1]. This is compounded by the rising prevalence of cardiovascular diseases (CVDs) – disorders affecting the heart and blood vessels – which are substantially more common in people with diabetes mellitus [1–4]. Both diabetes mellitus and CVDs are leading causes of morbidity and mortality worldwide, with the burden heaviest in LMICs [1], and CVDs represent the primary cause of death among people living with diabetes mellitus [1,5–8]. Despite this dual burden and its severe consequences for individuals and already strained health systems, limited research has quantified the composite risk of CVD, based on key factors, among people with diabetes mellitus [3–5,9].

Diabetes mellitus and CVDs share similar risk factors such as smoking, elevated blood pressure, and obesity, which increase the likelihood of comorbidity [6–8]. When these conditions coexist, they significantly increase the risk of mortality, complicate treatment through polypharmacy and associated risks like drug interactions and adverse reactions, and reduce overall quality of life [5,7,8,10,11]. The burden extends beyond individuals, driving higher long-term healthcare utilization and costs. These concerns are especially pronounced in LMICs, where noncommunicable diseases (NCDs) are often underdiagnosed or detected only at advanced stages, deaths are disproportionately high, and healthcare resources, insurance coverage, and operational NCD policies are limited [5,7,8].

Occurring independently or comorbidly, diabetes mellitus and CVD are a clear development drag for LMICs, many of which are African countries. While there is growing literature in African countries on identifying risk factors for diabetes mellitus and CVD individually [8,10], there is still a paucity of research quantifying the composite risk of CVD in people living with diabetes mellitus. The increasing risk of CVD is fueled by the cumulative interaction of risk factors [10], and this synergy between risk factors may worsen outcomes in people already living with diabetes mellitus. This study, therefore, aimed to address this knowledge gap by characterizing and quantifying the risk of CVD comorbidity in people living with diabetes mellitus. Specifically, the objective of this study was to examine the association between sociodemographic characteristics and the composite risk of developing CVD among people living with diabetes mellitus in Africa. As both diabetes mellitus and CVD have gendered effects and manifestations, we additionally investigated the sex-specific relationship of sociodemographic factors and the composite CVD risk. Characterizing the risks associated with this vicious synergy will help in the proper targeting and prioritizing of preventive and curative care to improve the management of these NCDs.

## Subjects, materials, and methods

### Data source

Data for this study were obtained from the WHO STEPwise approach to NCD risk factor Surveillance (STEPS) [12]. This population-based, cross-sectional survey elicits information on respondents’ behavioral and sociodemographic qualities, biochemical and physical measurements, as well as their self-reported health status on specific NCD-related conditions. The STEPS survey is administered in more than 120 countries, 33 of which are African [12]. While the WHO provides a standardized questionnaire for the STEPS survey, countries are allowed to adapt this questionnaire to reflect their specific sociocultural needs and peculiarities. Therefore, data harmonization was performed to account for the variations in questions and response coding across the countries.

Data from the latest surveys administered were used, and countries that did not collect information on age, pregnancy status, diabetes diagnosis, and prior CVD diagnosis were excluded. A total of 27 African countries met these conditions and were included in the analysis. In addition, questionnaires and data were translated to English for countries where the survey was conducted in other languages. Because of the limitations of the translation software used (DeepL), only translations from French and Portuguese to English were possible. Where language translation was not possible, an earlier survey administered in English was used, if available. A map view of the countries (included and excluded), as well as a detailed description of the countries, including sample size, language, and year of survey, are provided in Figure S1 and Table S1, Supplemental digital content 1, https://links.lww.com/CAEN/A74.

### Study population

Nonpregnant adults between 18 and 69 years of age who had diabetes and who did not self-report a prior CVD diagnosis were included in the study population. Individuals were identified as having diabetes if they self-reported a diagnosis by a health professional or had fasting blood glucose levels exceeding 7 mmol/L or 126 mg/dl at the time of the survey.

### Variables of interest

#### Predictors

Six sociodemographic factors were included as predictors in this study. These were sex assigned at birth, age of the respondent at the time of the survey, highest level of education achieved, current marital status, occupation, and household size. *Education,* defined as the highest level of education achieved, was categorized into ‘no formal education’, ‘less than secondary school completed’, ‘secondary education completed’, ‘baccalaureate/college completed/higher education’, ‘postgraduate education’, ‘religious education’. *Marital status* had three categories: ‘single/never married’, ‘married or cohabiting’, and ‘widowed or separated from partner’; and *occupation,* based on the respondents’ main work status in the 12 months preceding the survey, was grouped into being ‘employed’, ‘unemployed’, a ‘student’, or ‘not in the labor force’ (including those who were retired, disabled, or unable to work for other reasons). *Age* (in years) and *household size* were left as continuous variables.

#### Outcome

We created the STEPS-CARDIO index to quantify the risk of CVD, using key CVD risk factors including blood pressure, diet, and other lifestyle factors [2,8,10]. This index, an adaptation of the CANHEART health index [2], assigned participants a score of 1 for nonideal behaviors, like smoking, and 0 for the opposite. In other words, for each of the five risk factors/metrics [smoking, hypertension, recreational physical activity, diet (fruits and vegetable consumption), and obesity/overweight], each respondent received a score of 1 for nonideal behaviors, such as smoking, or 0 for practicing or reporting a public health recommended/approved behavior (Table S2, Supplemental digital content 1, https://links.lww.com/CAEN/A74). Respondents then received a STEPS-CARDIO risk index ranging from 0 (lowest risk) to 5 (highest risk), based on the sum of the assigned scores. The definitions of the included risk factors align with the CANHEART index and the WHO lifestyle recommendations [2,13].

### Statistical analysis

To quantify the risk of CVD comorbidity in people living with diabetes mellitus, the STEPS-CARDIO index was calculated for each respondent, and country-level summaries of the composite CVD risk were estimated. For descriptive purposes, STEPS-CARDIO scores of '0-1', '2-3', and '4-5' are classified as 'low risk,' 'medium risk,' and 'high risk,' respectively. We then fitted multivariable ordinal logistic regression models to assess the relationship between sociodemographic characteristics (listed in the ‘predictors’ section above) and CVD risk (STEPS-CARDIO index). Specifically, generalized linear mixed models for clustered ordinal responses models (package: ‘clmm’) were used, including a random intercept for country to account for the nonindependence of observations/respondents that share the same country [14]. An exchangeable covariance structure was assumed due to the spatial distribution of the clustering variable. The models were first run for the overall population, and then for sex-specific (women and men) subpopulations.

Geographic variation in demographic and health system characteristics may lead to differences in CVD risk among people with diabetes mellitus across Africa; therefore, we used mixed-effects models to estimate the average association across all observations, irrespective of their country. We also assessed whether observed patterns were consistent across African regions by grouping the countries into five regions (Central, East, North, South, and West Africa) [15], and repeating the analysis within each region, with countries maintained as random intercepts. All statistical analyses were performed with R, version 2024.04.2 + 764 (Posit Software).

#### Missing data

**Fig. 1:**
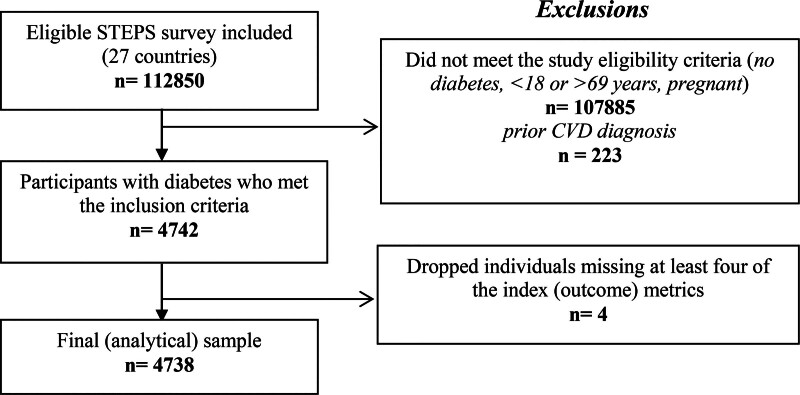
Flowchart of study population selection. CVD, cardiovascular disease.

Individuals with missing data on more than three index metrics were excluded (*n* = 4; Fig. 1). For the remaining individuals, missing values in both the index metrics and sociodemographic predictors (Tables S2 and S3, Supplemental digital content 1, https://links.lww.com/CAEN/A74) were addressed using multiple imputations by chained equations, which produced 50 imputed datasets, each generated through 30 iterations [16]. The primary analyses were then conducted using these imputed datasets, and regression results from each dataset were pooled to produce the final estimates.

#### Sensitivity analyses

Although there are mixed opinions about imputing missing outcome data, various papers have imputed outcome data and found that this method did not substantially change the results, compared to complete case analysis [17]. Nonetheless, the following sensitivity analyses were conducted to assess the robustness of the primary analyses performed on the imputed datasets, which included imputed values for missing outcome index metrics: (a) excluding the index metric with the most missingness (elevated blood pressure) from the STEPS-CARDIO score computation (Table S3, Supplemental digital content 1, https://links.lww.com/CAEN/A74), and (b) excluding anyone with missing data on any of the index metrics (‘index complete case analysis’) (Table S4, Supplemental digital content 1, https://links.lww.com/CAEN/A74).

### Ethics approval

This study used anonymized secondary data from the WHO, so ethical clearance was not required; however, we maintained high data security by encrypting the data and restricting access to designated team members throughout the research process.

## Results

### Descriptive analysis

A total of 112 850 individuals were surveyed in the 27 included African countries. Of these, 4738 (4.2%) individuals either self-reported a diabetes diagnosis or had fasting blood glucose levels exceeding 7 mmol/L (126 mg/dl) and met the other study eligibility criteria, forming the final analytical sample (Fig. 1). This proportion aligns closely with reported diabetes prevalence rates in Africa, which range between 4.2 and 5% [1,18].

Algeria (15.9%) and Morocco (13.9%) contributed the most to the sample size, while Madagascar had the least number of respondents (0.57%) (Table S1, Supplemental digital content 1, https://links.lww.com/CAEN/A74). The sampled population had a mean age of 46.84 years and included more women (*n* = 2660, 56%) than men. There was a preponderance of respondents who had no or less than secondary education (*n* = 3369, 71.1%). Similarly, most of the respondents were married (*n* = 2881, 61.2%) and employed (*n* = 2673, 56.4%) (Table 1).

**Table 1:** Sociodemographic characteristics of study population

Variable	Categories	*n* (%) or mean (SD) (*n* = 4738)
Age (years)		46.84 (12.9)
Sex	Men	2078 (44%)
	Women	2660 (56%)
Education (highest level)	No formal education	1581 (33.4%)
	Less than secondary school completed/attended	1788 (37.7%)
	Secondary education completed	662 (14%)
	Baccalaureate/college completed/higher education	442 (9.3%)
	Postgraduate education	103 (2.2%)
	Religious education	154 (3.3%)
	Missing	8 (0.2%)
Marital status	Single/never married	399 (8.4%)
	Married/common law/cohabitation	2881 (61.2%)
	Widowed/separated/divorced	648 (14.1%)
	Missing	810 (16.3%)
Occupation^a^	Employed	2673 (56.4%)
	Unemployed	1533 (32.4%)
	Student	64 (1.4%)
	Not in the labor force	418 (8.8%)
	Missing	50 (1.1%)
Household size		2.83 (1.8)

a Employed, included all employed positions/self-employment; unemployed, volunteer/housewife/unemployed; not in the labor force, retired/disabled/unable to work.

**Fig. 2:**
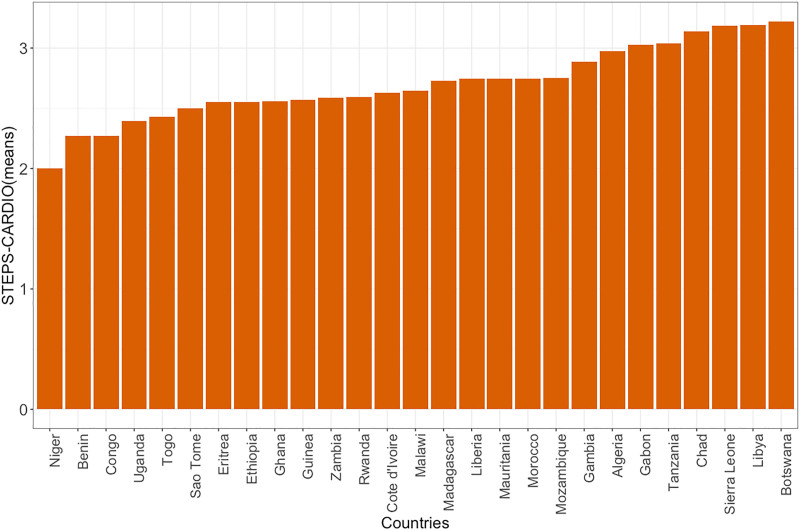
Country-level mean^†^ STEPS-CARDIO scores. ^†^Note that this is based on the mean of the country-level average STEPS-CARDIO index score across all (50) imputed datasets. Additional information is provided in Table S6, Supplemental digital content 1, https://links.lww.com/CAEN/A74.

Regarding the metrics used to estimate the STEPS-CARDIO index, most participants were nonsmokers (89.3%), overweight or obese (53.9%), consumed fewer than five servings of fruits and vegetables per day (72.8%), did not meet the recommended levels of weekly physical activity (92.9%), and about half (50.6%) reported having normal blood pressure (Table S5, Supplemental digital content 1, https://links.lww.com/CAEN/A74). The mean STEPS-CARDIO score ranged from 2 to 3.22, signifying that, on average, people with diabetes in the included countries had a medium risk of developing CVD (Fig. 2 and Table S6, Supplemental digital content 1, https://links.lww.com/CAEN/A74). Of all the countries, Botswana had the highest mean STEPS-CARDIO score (3.22), while Niger had the lowest mean (2.00).

### Risk of cardiovascular disease in people living with diabetes

**Fig. 3:**
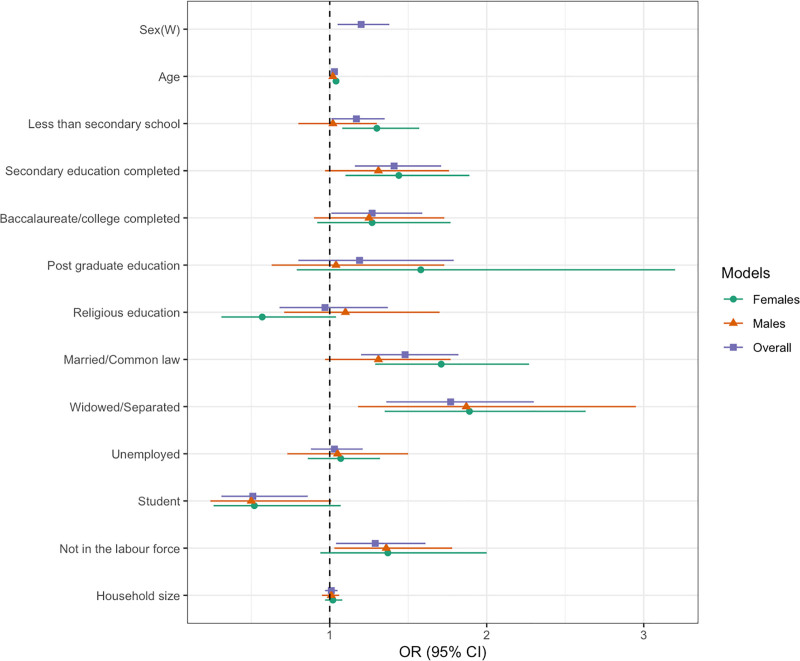
Odds ratio plot of factors associated with elevated diabetes mellitus-CVD comorbidity in Africa, for the overall and sex-specific models. Reference groups: sex, men; education, no formal education; marital status, single; occupation, employed. CI, confidence interval; OR, odds ratio.

Figure 3 and Table S7, Supplemental digital content 1, https://links.lww.com/CAEN/A74 show the relationship between the risk of CVD and individual sociodemographic factors in people with diabetes mellitus, both overall and stratified by sex. Older age was strongly and consistently associated with higher CVD risk across all models: each additional year increased the likelihood of a higher CVD risk by 3% [odds ratio (OR): 1.03, 95% confidence interval (CI): 1.02–1.04], a finding consistent across men and women. Sex differences were also evident as women had 20% higher odds of elevated CVD risk compared with men (OR: 1.20, 95% CI: 1.05–1.38).

Other sociodemographic characteristics showed mixed associations. Compared with individuals without formal education, those with higher levels of education were more likely to have increased CVD risk. In contrast, respondents with religious education appeared to have a minimal, nonsignificant protective association (OR: 0.97, 95% CI: 0.68–1.37). Marital or partnership status and household size were also associated with higher CVD risk, but increasing household size showed no significant relationship. Overall, these associations were broadly consistent across men and women, with a few notable exceptions: secondary education (attendance or completion) was significantly associated with higher CVD risk only among women. While being married or separated was associated with higher CVD risk in both sexes, the association for current partnership reached statistical significance only among women as well (OR: 1.71, 95% CI: 1.29–2.27). Lastly, religious education appeared protective for women but not for men (OR: 0.57, 95% CI: 0.31–1.04 vs. OR: 1.10, 95% CI: 0.71–1.70).

Occupational status was associated with increased CVD risk to varying levels. Compared with employed individuals, those not in the labor force (including retirees and other groups unable to work) showed higher CVD risk, though this association was not statistically significant for women. On the other hand, being a student was protective (OR: 0.51, 95% CI: 0.31–0.86) across all models. Recognizing that students are typically younger, and that most students in our sample were less than 30 years old (Table S8, Supplemental digital content 1, https://links.lww.com/CAEN/A74), we examined whether this association was age-driven by including a product term of age and occupation in the models. This analysis confirmed that age modified the relationship. Specifically, at the baseline age, students were considerably less likely than employed individuals to have elevated CVD risk (OR: 0.07, 95% CI: 0.01–0.46); however, this protective association decreases with age: for each additional year of age, students’ odds of elevated CVD risk increased by 11% (1.03 × 1.08 = 1.11), indicating that the advantage associated with being a student diminishes as they get older (Table S9, Supplemental digital content 1, https://links.lww.com/CAEN/A74).

Region-stratified analyses largely mirrored the primary overall results in direction, though effect sizes varied across regions. For example, the association between higher formal education and CVD risk was stronger in Central Africa than in other regions or the overall population (Table S10, Supplemental digital content 1, https://links.lww.com/CAEN/A74). Most within-region associations did not reach statistical significance, likely because of smaller sample sizes, which ranged from 168 to 1928 per stratum. Notably, no variables showed a statistically significant reversal of effect direction compared with the overall analysis, suggesting general consistency of associations across African regions.

### Sensitivity analyses

The results of the sensitivity analyses were generally consistent with the primary analyses, except for ‘not in the labor force’ and ‘being a student’ (Table S11 and Figure S2, Supplemental digital content 1, https://links.lww.com/CAEN/A74). In the first sensitivity analysis, where elevated blood pressure was excluded from the index calculation, the ORs were smaller than in the primary analysis, and the protective association for students was observed across both sexes. This suggests that hypertension is an important contributor to the diabetes mellitus–CVD comorbidity risk captured by the STEPS-CARDIO index, and its exclusion may underestimate overall risk. On the other hand, the second sensitivity analysis (‘index complete case analysis’) produced estimates largely comparable to the primary analysis, except that student status was no longer protective in men. As expected, CIs were wider in the index complete case sensitivity analysis because of smaller sample sizes.

## Discussion

It is well-established that the risk of CVD is much increased in people with diabetes mellitus [8,19]; however, an understanding of the extent of such CVD risk elevation among people with diabetes in Africa, as well as its association with their sociodemographic characteristics, is sparse. Our study is thus important as it addresses this gap and provides insights on the composite risk of CVD for patients with diabetes. In this multinational African study, we found that people living with diabetes mellitus had a medium risk of developing CVD, and that the composite risk of CVD was higher for older adults, women, as well as people with higher education, not in the labor force, or who were currently or previously married.

Consistent with other studies that highlight an increased CVD risk in aging diabetes populations, age was a strong predictor of CVD risk for both men and women in our sample [8,20–22]. This may be because of vascular aging, cumulative exposure, and manifestation of risk factors in older ages, and likely longer diabetes duration [22,23]. With increasing aging populations worldwide, this raises concerns about the diabetes mellitus–CVD comorbidity presenting more prominently at older ages, when individuals are more frail and immunocompromised, and directly threatens the maintenance of a high quality of life at old age.

Our study also noted sex differences in CVD risk among people with diabetes mellitus. Women in the surveyed population had a lower diabetes mellitus prevalence compared with men (4.2 vs. 4.6%; Table S12, Supplemental digital content 1, https://links.lww.com/CAEN/A74), but showed a 20% higher likelihood of diabetes mellitus–CVD comorbidity [24,25]. This is consistent with previous studies indicating that women are less likely than men to be diagnosed with diabetes until postmenopause, after which rates become comparable or even higher, yet they continue to experience greater CVD-related mortality when diagnosed, even at younger ages [19,20,22]. Although we were unable to distinguish between types of diabetes in our study, a previous study estimated that type 2 diabetes increases the risk of a cardiovascular event by 25–50% in women relative to men [24]. While biological factors, such as menopause-related changes [22], likely contribute to this sex difference, psychosocial, cultural, and behavioral factors are also important. Despite having greater CVD protective benefits from physical activity [20], adherence to the recommended physical activity guidelines is usually lower in women [2,13,24]. Inequities in healthcare provision that disadvantage women have also been documented [20]. In addition, persistent societal expectations for African women to serve as primary caregivers, despite improvements in female education and employment, may leave women having to juggle work and home responsibilities, which may then increase stress and contribute further to elevated CVD risk [26].

No significant association was observed between household size and CVD risk in our study, although individuals from larger households had slightly higher CVD risk, with the association being somewhat stronger in women. As mentioned earlier, caregiving and household responsibilities often fall on women in African communities, leading to psychosocial stress that can adversely impact their health [26,27]; however, at least one study has found a protective effect of household size on cardiovascular risk and outcomes, possibly because of the composition and age distribution of household members [27]. The study explained that household tasks may be shared among older household members, thereby reducing the burden typically placed on older women [27]. Nonetheless, it must be noted that lower socioeconomic status (SES) is linked to larger household size, and the underlying factors related to SES may be driving the higher CVD risk for people with larger households [28].

Socioeconomic stressors, such as those from lower education levels and unemployment, have been previously linked to increased CVD risk and poorer health outcomes [10,28,29]. However, contrary to the consensus that higher education is inversely associated with CVD risk [8,10,30], our study found that increasing formal education levels were associated with higher CVD risk instead. At least one other African study has reported similar findings [8]. Higher education is generally linked to lower smoking rates, better healthcare access, and higher income [10], but rising smoking rates among African youth [29,31] and high unemployment rates in many African countries [32] may limit the benefits of education on health. For example, even among educated individuals, cost-related barriers to healthcare may delay diabetes screening, diagnosis, and treatment [11], which – together with unemployment – may help explain the observed associations of both education and unemployment with increased CVD risk in our study [10,29,33]. It is also possible that this observation reflects missed diabetes cases in our study. Previous studies indicate that individuals with lower education levels have higher rates of undiagnosed diabetes [8], thus our restriction to individuals already diagnosed with diabetes may partly account for this finding as well.

An interesting observation from our study was that being a student appeared protective against CVD for both men and women. Students are usually grouped as ‘not being in the labor force’; a category often associated with higher CVD risk in the general population [34]; however, we classified students as a separate occupation category because many work while studying [35]. Additional analyses showed that most students in our sample were under 30 years (87.5%), and the interaction of age and occupation confirmed that age was a partial driver of the observed protective effect, consistent with the well-established increase in CVD risk with age [8,20,21,23]. Nevertheless, diabetes mellitus and CVD prevalence is rising among younger populations [1,36], and screening strategies targeted at these groups to ensure earlier detection and management of these conditions are necessary.

## Strengths and limitations

This study is among the first to quantify CVD risk using a composite index among people with diabetes mellitus in Africa, with curated data from a nationally representative survey administered across multiple African countries. Our use of a composite index allowed us to observe the collective impact of major CVD risk factors, while also providing insights into how the risk varies between men and women, particularly given the gendered manifestation of diabetes mellitus and CVDs. The observed associations remain informative, even though the composite nature of the STEPS-CARDIO index makes it difficult to pinpoint which behaviors/metrics contributed the most to the score. While imputation of some metrics may have reduced precision [17], the sensitivity analyses conducted support the robustness of our findings. The index does not capture other important CVD risk factors, such as sleep health and blood lipid levels [10], as they were either sparsely collected across countries or not included in the survey. Because of similar data limitations, other predictors of the diabetes mellitus–CVD comorbidity risk, such as diabetes duration, glycemic control, medication use, and healthcare utilization were not included in our analyses as well. In addition, our study is descriptive and aimed at identifying associations between social factors and CVD risk rather than establishing causal relationships.

While supplementing self-reported diabetes with laboratory measurements captured more cases, these biochemical data were available in only a few countries, so the sample likely does not fully represent all diabetes cases. We were also unable to distinguish between diabetes types. Although the predictors and index metrics included in the study were self-reported and may be prone to recall or reporting bias, they remain the most reliable approach for assessing diabetes burden in population-based surveys in LMICs [2,37,38]. Further, cross-country comparisons are limited, as we combined survey data from a 7-year period without accounting for time trends. The high representation of North African adults in our sample (41%), combined with geographic heterogeneity in demographic and other factors across Africa, could have influenced our results. To account for this, we included a random intercept for country in all models and conducted regional analyses, which were generally consistent with the overall findings, suggesting that associations were similarly directed across regions. Nevertheless, our results are based on 27 countries and may not be fully generalizable to the entire continent. More comprehensive and standardized data collection across all African regions is needed to improve the representativeness and generalizability of future analyses.

### Conclusion

This study offers a comprehensive view of the CVD risk among adults with diabetes in Africa, showing that most are at medium risk, with variations in risk levels across sex and socioeconomic classes. This increasing likelihood of diabetes mellitus–CVD comorbidity reflects the ongoing shift in Africa’s disease burden from communicable to NCDs, with serious implications for individuals at risk and already strained healthcare systems. It, thus, underscores the urgent need for culturally tailored, patient-centered, multidisciplinary interventions that actively engage patients, healthcare professionals, and community leaders.

Once diagnosed with diabetes mellitus, patients should receive a care plan that is sensitive to their health history, specific needs, and social contexts. This plan should include regular CVD screening, patient education, and self-management support. As effective diabetes mellitus management involves consistent and timely use of primary healthcare (PHC) services [1], better integration of NCD care into PHC in Africa is needed to facilitate routine monitoring. However, as PHC is often underutilized as the primary point of contact in many African countries [31,39], community-based NCD screening and management programs – including mobile clinics, outreach initiatives, and home visits, – should be strengthened and made more consistent to improve continuity of care for people with diabetes mellitus. In addition, peer-led interventions, such as walking groups, can promote physical activity and other risk management strategies among women and other high-risk groups. Given that social and community groups – such as age-grade associations, women’s cooperatives, faith-based organizations, and savings or rotating credit groups – are common in Africa [40,41], such interventions can be integrated into the regular activities of these groups to enhance participation and promote the health of their members. These strategies can be implemented alongside broader efforts to strengthen health system capacity and optimize resource allocation, ensuring more effective screening and management of diabetes mellitus–CVD comorbidity.

## Acknowledgements

We thank the Data Archives of the WHO for prompt approval of access to the NCD Microdata Repository.

This research was primarily enabled by funding from the McGill Global Noncommunicable Diseases Microgrant Program.

R.I.E-N. conceptualized and designed the study. D-F.C.O., H.O., and U.V.U. helped in the literature search. D-F.C.O., H.O., S.C.O., and U.V.U. developed the analysis plan. D-F.C.O. and R.I.E-N. carried out the statistical analysis. All authors jointly drafted, reviewed, and approved the manuscript for submission.

The findings from this study were shared during a poster presentation at the 4^th^ Annual Black Excellence in Science, Technology, Engineering, Mathematics and Medicine (BE-STEMM) meeting in Ottawa, Canada; July 2024.

### Conflicts of interest

There are no conflicts of interest.

## Supplementary Material
