# Supplementary material for: Composite risk of cardiovascular disease comorbidity in people living with diabetes in Africa
Source: Cardiovasc Endocrinol Metab. 2025 Oct 16;14(4):e00347. doi: 10.1097/XCE.0000000000000347 (PMC12534003; doi:10.1097/XCE.0000000000000347)
Supplement: Supplementary file 1 [file xce-14-e00347-s001.docx]

**Full title:** Composite risk of cardiovascular disease comorbidity in people living with diabetes in Africa

**Short title:** Diabetes-CVD comorbidity risk in Africa

**Authors:** Regina Idu EJEMOT-NWADIARO^a, b, c^, Divine-Favour OFILI^d^, Stephen Chukwuma OGBODO^d, e^, Henshaw OKOROIWU^f, g^, Ugochinyere Vivian UKAH^d,h^

^a^ Department of Public Health, School of Allied Health Sciences, Kampala International University, Ishaka, Uganda.

^b^ Directorate of Research, Innovation, Consultancy, and Extension, Kampala International University, Ishaka, Uganda.

^c^ Department of Public Health, Faculty of Allied Medical Sciences, University of Calabar, Calabar, Nigeria.

^d^ Department of Epidemiology, Biostatistics and Occupational Health, Faculty of Medicine and Health Sciences, McGill University, Montreal, Quebec, Canada.

^e^ Lady Davis Institute for Medical Research, Jewish General Hospital, Montreal, Quebec, Canada.

^f^ Department of Medical Laboratory Sciences, David Umahi Federal University of Health Sciences, Uburu, Ebonyi State, Nigeria.

^g^ International Institute for Oncology and Cancer Research, David Umahi Federal University of Health Sciences, Uburu, Ebonyi State, Nigeria.

^h^ Department of Medicine, Faculty of Medicine and Health Sciences, McGill University, Montreal, Quebec, Canada.

**Supplementary Materials**

**Figure S1.** Map view of countries included in the analysis

**Table S1.** Country distribution by STEPS survey sample size and study sample size

**Table S2.** Definition of the metrics for the STEPS-CARDIO index (outcome variable)

**Table S3.** Proportion of missing data per variable of interest

**Table S4.** Number of persons with missing index metrics (outcome) data (sum of missing metrics)

**Table S5.** Baseline distribution of the indicators for the STEPS-CARDIO index

**Table S6.** Country-level mean STEPS-CARDIO score (and standard deviation) across imputed datasets

**Table S7.** Ordinal logistic regression outputs (odds ratio and 95% CI) for the overall and sex-specific samples

**Table S8.** Distribution of students by age groups

**Table S9.** Ordinal logistic regression outputs (odds ratio and 95% CI) for the overall and sex-specific samples- **with age and occupational status interacted**

**Table S10.** Region-specific ordinal logistic regression mixed effects model outputs (odds ratio and 95% CI)

**Table S11.** Ordinal logistic regression outputs (odds ratio and 95% CI) of the primary analysis and two sensitivity analyses models for the overall and sex-specific models

**Figure S2.** Odds ratio plot of the primary analysis and two sensitivity analyses models for the overall and sex-specific models

**Table S12.** Prevalence of diabetes in included STEPS survey datasets

Supplementary material: Composite risk of cardiovascular disease comorbidity in people living with diabetes in Africa

Regina Idu EJEMOT-NWADIARO, Divine-Favour OFILI, Stephen Chukwuma OGBODO, Henshaw OKOROIWU, Ugochinyere Vivian UKAH

**Figure S1.** Map view of countries included in the analysis


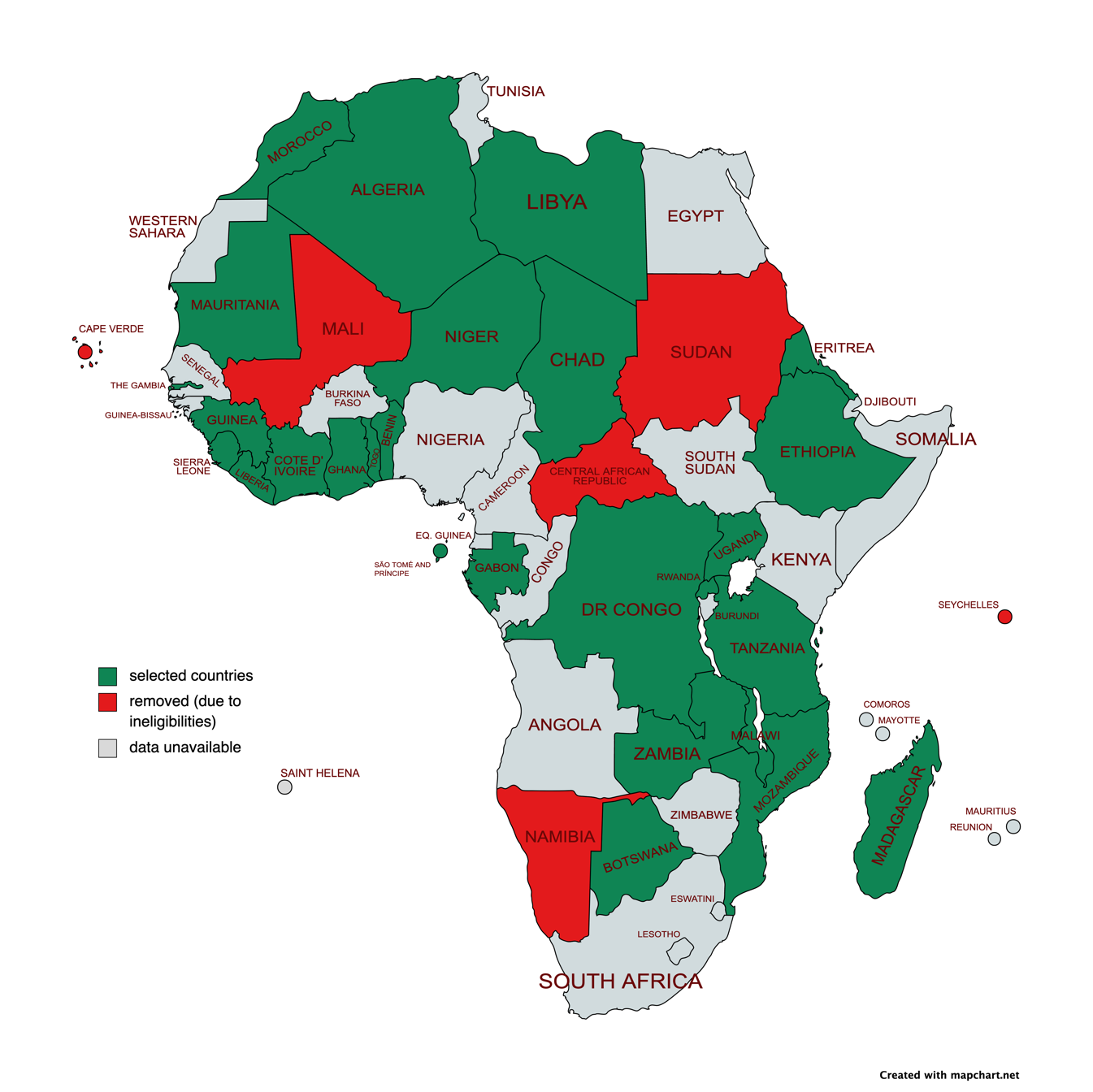
**Table S1.** Country distribution by STEPS survey sample size and study sample size

| **S/N** | **Country** | **Year** | **Language of questionnaire** | **STEPS Survey sample** | **Study sample** | **Region in Africa**^a^ |
| --- | --- | --- | --- | --- | --- | --- |
|  |  |  |  | **n= 112850** | **n = 4738** |  |
| 1 | Algeria | 2016 | French | 6989 | 753 | North Africa |
| 2 | Benin | 2015 | French | 5126 | 299 | West Africa |
| 3 | Botswana | 2014 | English | 4070 | 162 | South Africa |
| 4 | Chad | 2008 | French | 1971 | 35 | Central Africa |
| 5 | Cote d’Ivoire | 2005 | French | 4742 | 49 | West Africa |
| 6 | DR Congo | 2005 | English | 1943 | 48 | Central Africa |
| 7 | Eritrea | 2010 | English | 6265 | 197 | East Africa |
| 8 | Ethiopia | 2015 | English | 9800 | 247 | East Africa |
| 9 | Gabon | 2009 | French | 2708 | 34 | Central Africa |
| 10 | Gambia | 2010 | English | 4103 | 32 | West Africa |
| 11 | Ghana | 2006 | English | 2662 | 128 | West Africa |
| 12 | Guinea | 2009 | French | 2491 | 90 | West Africa |
| 13 | Liberia | 2011 | English | 2503 | 143 | West Africa |
| 14 | Libya | 2009 | English | 3590 | 434 | North Africa |
| 15 | Madagascar | 2005 | French | 5626 | 27 | South Africa |
| 16 | Malawi | 2017 | English | 4187 | 78 | South Africa |
| 17 | Mauritania | 2006 | French | 2600 | 82 | North Africa |
| 18 | Morocco | 2017 | French | 5429 | 659 | North Africa |
| 19 | Mozambique | 2005 | Portuguese | 3310 | 54 | South Africa |
| 20 | Niger | 2007 | French | 2757 | 473 | West Africa |
| 21 | Rwanda | 2012 | English | 7226 | 108 | East Africa |
| 22 | Sao Tome and Principe | 2008 | Portuguese | 2457 | 51 | Central Africa |
| 23 | Sierra Leone | 2009 | English | 4997 | 34 | West Africa |
| 24 | Togo | 2010 | French | 4370 | 94 | West Africa |
| 25 | Uganda | 2014 | English | 3987 | 55 | East Africa |
| 26 | United Republic of Tanzania^b^ | 2011 | English | 2639 | 93 | East Africa |
| 27 | Zambia | 2017 | English | 4302 | 279 | South Africa |

^a^ The region classification was based on the African Development Bank Group (https://www.afdb.org/en/countries)

^b^ The 2011 survey was used for Tanzania, because the latest version (2012) was in Kiswahili (a language not recognized in the translator software)

**Table S2:** Definition of the metrics for the STEPS-CARDIO index (outcome variable)^[2]^

| **Risk factor/metric** | **Definition of non-ideal behaviour (coded as ‘1’)** |
| --- | --- |
| Smoking | Current smoker or former smoker who quit smoking less than 12 months ago |
| Overweight/obesity | Body Mass Index (BMI) ≥ 25 |
| Elevated blood pressure | Self-reported diagnosis of elevated blood pressure by a health care professional |
| Physical activity | <150 minutes of moderate intensity physical activity like walking or swimming per week |
| Diet | <5 servings of fruit and vegetables per day |

**Table S3.** Proportion of missing data per variable of interest

| **Variable** ^†^ | **% missingness** |
| --- | --- |
| Age | .. |
| Sex | .. |
| Country | .. |
| Education (highest level) | 0.2 |
| Occupation | 1.1 |
| Marital status | 16.3 |
| Household size | 14.6 |
| Smoking | 0.1 |
| BMI | 0.8 |
| Elevated blood pressure (*self-reported*) | 21.2 |
| Leisure physical activity | 0.9 |
| Fruit and vegetable consumption | 4 |

†*All the listed variables were included in the imputation model*

**Table S4.** Number of persons with missing index metrics (outcome) data (sum of missing metrics)

| **Missing data (index metrics)** | **Frequency** |
| --- | --- |
| 0 | 3513 |
| 1 | 1173 |
| 2 | 49 |
| 3 | 3 |
| 4 | 3 |
| 5 | 1 |

**Table S5.** Baseline distribution of the indicators for the STEPS-CARDIO index

| **Variable** | **Categories** | **Overall sample**  **n (%)**  **n=4738** | **Women only**  **n (%)**  **n=2660** | **Men only**  **n (%)**  **n= 2078** |
| --- | --- | --- | --- | --- |
| Smoking | Yes (1) | 503 (10.6%) | 55 (2.1%) | 448 (21.6%) |
|  | No (0) | 4232 (89.3%) | 2604 (97.9%) | 1628 (78.3%) |
|  | Missing | 3 (0.1%) | 1 (0.04%) | 2 (0.1%) |
| BMI | ≥ 25 (1) | 2557 (54%) | 1634 (61.4%) | 923 (44.4%) |
|  | <25 (0) | 2142 (45.2%) | 1008 (21.3%) | 1134 (54.6%) |
|  | Missing | 39 (0.8%) | 18 (0.7%) | 21 (1.0%) |
| Elevated blood pressure (*self-reported*) | Yes (1) | 1336 (29.2%) | 890 (33.5%) | 446 (21.5%) |
|  | No (0) | 2397 (50.6%) | 1291 (48.5%) | 1106 (53.2%) |
|  | Missing | 1005 (21.2%) | 479 (18%) | 526 (25.3%) |
| Physical activity | <30 mins (1) | 4403 (92.9%) | 2521 (94.8%) | 1882 (90.6%) |
|  | ≥30 mins (0) | 288 (6.1%) | 123 (4.6%) | 165 (7.9%) |
|  | Missing | 47 (1%) | 16 (0.6%) | 31 (1.5%) |
| Fruit and vegetable consumption | <5 servings (1) | 3447 (72.8%) | 1935 (72.7%) | 1512 (72.8%) |
|  | ≥ 5 servings (0) | 1103 (23.3%) | 638 (24%) | 465 (22.4%) |
|  | Missing | 188 (4%) | 87 (3.3%) | 101 (4.9%) |

See the Appendix 1 for definition of the index variables as culled from (Maclagan et al; 2014)^[2]^

**Table S6.** Country-level mean STEPS-CARDIO score (and standard deviation) across imputed datasets

| **Country** | **Mean of means** | **Sd of means** |
| --- | --- | --- |
| Algeria | 2.98 | 0.01 |
| Benin | 2.27 | 0.02 |
| Botswana | 3.22 | 0.01 |
| Chad | 3.14 | 0.05 |
| Congo | 2.27 | 0.00 |
| Cote d'Ivoire | 2.63 | 0.00 |
| Eritrea | 2.55 | 0.02 |
| Ethiopia | 2.55 | 0.03 |
| Gabon | 3.03 | 0.00 |
| Gambia | 2.88 | 0.01 |
| Ghana | 2.56 | 0.01 |
| Guinea | 2.57 | 0.04 |
| Liberia | 2.75 | 0.02 |
| Libya | 3.19 | 0.01 |
| Madagascar | 2.73 | 0.08 |
| Malawi | 2.65 | 0.05 |
| Mauritania | 2.75 | 0.03 |
| Morocco | 2.75 | 0.01 |
| Mozambique | 2.75 | 0.04 |
| Niger | 2.00 | 0.01 |
| Rwanda | 2.59 | 0.04 |
| Sao Tome | 2.50 | 0.01 |
| Sierra Leone | 3.19 | 0.05 |
| Tanzania | 3.04 | 0.02 |
| Togo | 2.43 | 0.03 |
| Uganda | 2.39 | 0.05 |
| Zambia | 2.59 | 0.02 |

**Table S7**. Ordinal logistic regression outputs (odds ratio and 95% CI) for the overall and sex-specific samples

| **Variable/ models** | | **Overall \| (n=**4738**)**  **OR (95% CI)** | **Women only \| (n =**2660)  **OR (95% CI)** | **Men only \| (n** = 2078)  **OR (95% CI)** |
| --- | --- | --- | --- | --- |
| Age | .. | 1.03 (**1.02- 1.04**) | 1.04 (**1.03, 1.04**) | 1.02 (**1.01, 1.03**) |
| Sex | Men (ref) | .. | .. | .. |
|  | Women | 1.20 (**1.05 - 1.38**) | .. | .. |
| Education (highest level) | 1- No formal education (ref) | .. | .. | .. |
|  | 2- Less than secondary school completed/attended | 1.17 (**1.01, 1.35**) | 1.30 (**1.08, 1.57**) | 1.02 (0.80, 1.30) |
|  | 3- Secondary education completed | 1.41 (**1.16, 1.71**) | 1.44 (**1.10, 1.89**) | 1.31 (0.97, 1.76) |
|  | 4- Baccalaureate/college completed/higher education | 1.27 (**1.01, 1.59**) | 1.27 (0.92, 1.77) | 1.25 (0.90, 1.73) |
|  | 5- Post graduate education | 1.19 (0.80, 1.79) | 1.58 (0.79, 3.20) | 1.04 (0.63, 1.73) |
|  | 6- Religious education | 0.97 (0.68, 1.37) | 0.57 (0.31, 1.04) | 1.10 (0.71, 1.70) |
| Marital status | 1- Single/Never married (ref) | - | .. | .. |
|  | 2-Married/Common law/cohabitation | 1.48 (**1.20, 1.82**) | 1.71 (**1.29, 2.27**) | 1.31 (0.97, 1.77) |
|  | 3- Widowed/Separated/Divorced | 1.77 (**1.36, 2.30**) | 1.89 (**1.35, 2.63**) | 1.87 (**1.18, 2.95**) |
| Occupation | 1- Employed (ref) | .. | .. | .. |
|  | 2- Unemployed | 1.03 (0.88, 1.21) | 1.07 (0.86, 1.32) | 1.05 (0.73, 1.50) |
|  | 3- Student | 0.51 (**0.31, 0.86**) | 0.52 (0.26, 1.07) | 0.50 (0.24, 1.01) |
|  | 4- Not in the labour force | 1.29 (**1.04, 1.61**) | 1.37 (0.94, 2.00) | 1.36 (**1.03, 1.78**) |
| Household size | **-** | 1.01 (0.97, 1.05) | 1.02 (0.97, 1.08) | 1.01 (0.95, 1.06) |

*CI, Confidence intervals*; *OR, Odds ratio*

(CIs in bold are statistically significant)

**Table S8.** Distribution of students by age groups

| **Age group** | **n (%)** |
| --- | --- |
| Less than 30 years | 56 (87.5%) |
| 30-50 years | 6 (9.4%) |
| 50+ years | 2 (3.1%) |

**Table S9.** Ordinal logistic regression outputs (odds ratio and 95% CI) for the overall and sex-specific samples- with **age and occupational status interacted**

| **Variable/ models** | | **Overall \| (n=**4738**)**  **OR (95% CI)** | **Women only \| (n =**2660)  **OR (95% CI)** | **Men only \| (n** = 2078)  **OR (95% CI)** |
| --- | --- | --- | --- | --- |
| Age | .. | 1.03 (**1.02 – 1.03**) | 1.04 (**1.02 – 1.05**) | 1.02 (**1.01 – 1.03**) |
| Sex | Men (ref) | .. | .. | .. |
|  | Women | 1.19 (**1.04 – 1.37**) | .. | .. |
| Education (highest level) | 1- No formal education (ref) | .. | .. | .. |
|  | 2- Less than secondary school completed/attended | 1.18 (**1.02 – 1.37**) | 1.30 (**1.08 – 1.57**) | 1.02 (0.79 – 1.31) |
|  | 3- Secondary education completed | 1.41 (**1.15 – 1.72**) | 1.44 (**1.10 – 1.89**) | 1.28 (0.95 – 1.72) |
|  | 4- Baccalaureate/college completed/higher education | 1.27 (**1.01 – 1.59**) | 1.24 (0.89 – 1.74) | 1.24 (0.89 – 1.72) |
|  | 5- Post graduate education | 1.18 (0.78 – 1.78) | 1.57 (0.78 – 3.16) | 1.00 (0.59 – 1.70) |
|  | 6- Religious education | 0.97 (0.69 – 1.36) | 0.57 (0.31 – 1.05) | 1.09 (0.71 – 1.68) |
| Marital status | 1- Single/Never married (ref) | - | .. | .. |
|  | 2-Married/Common law/cohabitation | 1.46 (**1.20 – 1.79**) | 1.68 (**1.26 – 2.24**) | 1.29 (0.96 – 1.72) |
|  | 3- Widowed/Separated/Divorced | 1.73 (**1.33 – 2.26**) | 1.86 (**1.32 – 2.61**) | 1.80 (**1.11 – 2.93**) |
| Occupation | 1- Employed (ref) | .. | .. | .. |
|  | 2- Unemployed | 0.70 (0.42 – 1.17) | 0.97 (0.51 – 1.84) | 0.62 (0.17 – 2.24) |
|  | 3- Student | 0.07 (**0.01 – 0.46**) | 0.19 (**0.01 – 2.62**) | 0.03 (**0.002 – 0.38**) |
|  | 4- Not in the labour force | 2.32 (0.59 – 9.11) | 1.48 (0.20 – 10.78) | 3.31 (0.51 – 21.69) |
| Household size | .. | 1.01 (0.97 – 1.05) | 1.02 (0.97 – 1.07) | 1.01 (0.96 – 1.06) |
| Age x Occupation | Age x Employed (ref) | .. | .. | .. |
|  | Age x Unemployed | 1.01 (1.00 – 1.01) | 1.00 (0.99 – 1.02) | 1.01 (0.98 – 1.04) |
|  | Age x Student | 1.08 (**1.01 – 1.16**) | 1.04 (0.94 – 1.16) | 1.12 (**1.01 – 1.24**) |
|  | Age x Not in the labour force | 0.99 (0.97 – 1.01) | 1.00 (0.97 – 1.03) | 0.99 (0.95 – 1.02) |

*CI, Confidence intervals*; *OR, Odds ratio*

(CIs in bold are statistically significant)

**Table S10.** Region-specific ordinal logistic regression mixed effects model outputs (odds ratio and 95% CI)

| **Variable/ models** | | **Overall \| (n=**4738**)**  **OR (95% CI)** | **Central Africa \| (n=**168**)**  **OR (95% CI)** | **East Africa \| (n =**700)  **OR (95% CI)** | **North Africa \| (n** = 1928)  **OR (95% CI)** | **South Africa \| (n** = 600)  **OR (95% CI)** | **West Africa \| (n** = 1342)  **OR (95% CI)** |
| --- | --- | --- | --- | --- | --- | --- | --- |
| Age | .. | 1·03 (**1·02- 1·04**) | 1.07 (**1.03 – 1.10**) | 1.04 (**1.02 – 1.05**) | 1.03 (**1.02 – 1.04**) | 1.04 (**1.02 – 1.05**) | 1.02 (**1.01 – 1.03**) |
| Sex | Men (ref) | .. | .. | .. | .. | .. | .. |
|  | Women | 1·20 (**1·05 - 1·38**) | 1.82 (0.92 – 3.60) | 1.14 (0.80 – 1.62) | 1.05 (0.79 – 1.38) | 1.35 (0.95 – 1.92) | 1.31 (**1.04 – 1.66**) |
| Education (highest level) | 1- No formal education (ref) | .. | .. | .. | .. | .. | .. |
|  | 2- Less than secondary school completed/attended | 1·17 (**1·01, 1·35**) | 2.22 (0.86 – 6.47) | 1.58 (**1.10 – 2.27**) | 1.00 (0.79 – 1.27) | 1.18 (0.73 – 1.92) | 1.17 (0.89 – 1.55) |
|  | 3- Secondary education completed | 1·41 (**1·16, 1·71**) | 3.39 (**1.02 – 11.25**) | 2.86 (**1.59 – 5.14**) | 1.04 (0.78 – 1.38) | 1.43 (0.73 – 1.92) | 1.56 (**1.05 – 2.32**) |
|  | 4- Baccalaureate/college completed/higher education | 1·27 (**1·01, 1·59**) | 3.36 (0.91 – 12.35) | 2.00 (0.75 – 5.35) | 1.04 (0.76 - 1.44) | 1.27 (0.67 – 2.41) | 1.05 (0.64 – 1.73) |
|  | 5- Post graduate education | 1·19 (0·80, 1·79) | 1.72 (0.20 – 15.11) | 2.70 (0.47 – 15.43) | 0.74 (0.43 – 1.28) | 1.55 (0.27 – 9.03) | 2.00 (0.88 – 4.58) |
|  | 6- Religious education | 0·97 (0·68, 1·37) | 1.04 (0.10 – 10.95) | .. | 1.33 (0.57 – 3.08) | .. | 0.91 (0.61 – 1.36) |
| Marital status | 1- Single/Never married (ref) | - | .. | .. | .. | .. | .. |
|  | 2-Married/Common law/cohabitation | 1·48 (**1·20, 1·82**) | 1.61 (0.51 – 5.10) | 1.32 (0.69 – 2.51) | 1.95 (**1.37 – 2.77**) | 1.20 (0.73 – 1.95) | 1.27 (0.89 – 1.80) |
|  | 3- Widowed/Separated/Divorced | 1·77 (**1·36, 2·30**) | 1.44 (0.31 – 6.77) | 1.75 (0.84 – 3.64) | 2.26 (**1.45 – 3.53**) | 1.51 (0.84 – 2.72) | 1.69 (0.99 – 2.87) |
| Occupation | 1- Employed (ref) | .. | .. | .. | .. | .. | .. |
|  | 2- Unemployed | 1·03 (0·88, 1·21) | 1.50 (0.60 – 3.77) | 1.40 (0.90 – 2.17) | 0.91 (0.69 – 1.22) | 0.84 (0.58 – 1.21) | 1.39 (0.95 – 2.03) |
|  | 3- Student | 0·51 (**0·31, 0·86**) | 1.93 (0.44 – 8.46) | 0.20 (0.02 – 1.61) | 0.77 (0.32 – 1.88) | 0.46 (0.14 – 1.46) | 0.31 (**0.12 – 0.80**) |
|  | 4- Not in the labour force | 1·29 (**1·04, 1·61**) | 0.69 (0.22 – 2.15) | 1.42 (0.49 – 4.17) | 1.05 (0.77 – 1.44) | 1.21 (0.72 – 2.04) | 1.81 (**1.08 – 3.02**) |
| Household size | .. | 1·01 (0·97, 1·05) | 0.92 (0.77 – 1.10) | 1.08 (0.97 – 1.20) | 1.01 (0.96 – 1.07) | 1.02 (0.90 – 1.16) | 1.00 (0.94 – 1.06) |

*CI, Confidence intervals*; *OR, Odds ratio*

(CIs in bold are statistically significant)

Note: For all regions except South Africa, results were pooled from the 50 imputed datasets. In the South African dataset, one imputed dataset lacked a defined variance-covariance matrix and was therefore excluded from the pooling step.

**Table S11.** Ordinal logistic regression outputs (odds ratio and 95% CI) of the primary analysis and two sensitivity analyses models for the overall and sex-specific models

| **Variable/ models** | | **Primary analyses** | | | **Sensitivity analysis 1** | | | **Sensitivity analysis 2** | | | |
| --- | --- | --- | --- | --- | --- | --- | --- | --- | --- | --- | --- |
|  |  | **Overall \| (n=**4738**)**  **OR (95% CI)** | **Women only \| (n =**2660)  **OR (95% CI)** | **Men only \| (n** = 2078)  **OR (95% CI)** | **Overall \| (n=**4738**)**  **OR (95% CI)** | **Women only \| (n =**2660)  **OR (95% CI)** | **Men only \| (n** = 2078)  **OR (95% CI)** | **Overall \| (n=**3513**)**  **OR (95% CI)** | **Women only \| (n =**2076)  **OR (95% CI)** | **Men only \| (n** = 1437)  **OR (95% CI)** |  |
| Age | .. | 1.03 (**1.02- 1.04**) | 1.04 (**1.03, 1.04**) | 1.02 (**1.01, 1.03**) | 1.01 (**1.01-1.02**) | 1.02 (**1.01, 1.03**) | 1.00 (1.00-1.02) | 1.03 (**1.02-1.04**) | 1.04 (**1.03, 1.05**) | 1.02 (**1.01, 1.03**) |  |
| Sex | Men (ref) | .. | .. | .. | .. | .. | .. | .. | .. | .. |  |
|  | Women | 1.20 (**1.05 - 1.38**) | .. | .. | 1.00 (0.87, 1.14) | .. | .. | 1.18 (**1.01, 1.38**) | .. | .. |  |
| Education (highest level) | 1- No formal education (ref) | .. | .. | .. | .. | .. | .. | .. | .. | .. |  |
|  | 2- Less than secondary school completed/attended | 1.17 (**1.01, 1.35**) | 1.30 (**1.08, 1.57**) | 1.02 (0.80, 1.30) | 1.14 (0.99, 1.33) | 1.32 (**1.07, 1.57**) | 0.97 (0.76, 1.22) | 1.13 (0.95, 1.33) | 1.25 (**1.02, 1.54**) | 0.94 (0.70, 1.27) |  |
|  | 3- Secondary education completed | 1.41 (**1.16, 1.71**) | 1.44 (**1.10, 1.89**) | 1.31 (0.97, 1.76) | 1.49 (**1.22, 1.82**) | 1.62 (**1.22, 2.14**) | 1.27 (0.94, 1.71) | 1.28 (**1.03, 1.59**) | 1.38 (**1.03, 1.85**) | 1.09 (0.77, 1.56) |  |
|  | 4- Baccalaureate/college completed/higher education | 1.27 (**1.01, 1.59**) | 1.27 (0.92, 1.77) | 1.25 (0.90, 1.73) | 1.29 (**1.02, 1.62**) | 1. 47 (**1.03, 2.08**) | 1.13 (0.82, 1.56) | 1.14 (0.89, 1.46) | 1.16 (0.82, 1.65) | 1.07 (0.73, 1.58) |  |
|  | 5- Post graduate education | 1.19 (0.80, 1.79) | 1.58 (0.79, 3.20) | 1.04 (0.63, 1.73) | 1.16 (0.76, 1.77) | 1.75 (0.85, 3.61) | 0.93 (0.55, 1.57) | 1.26 (0.81, 1.97) | 1.86 (0.89, 3.89) | 1.02 (0.57, 1.82) |  |
|  | 6- Religious education | 0.97 (0.68, 1.37) | 0.57 (0.31, 1.04) | 1.10 (0.71, 1.70) | 0.88 (0.62, 1.25) | 0.49 (**0.27, 0.88**) | 1.05 (0.68, 1.62) | 1.03 (0.71, 1.51) | 0.60 (0.32, 1.15) | 1.16 (0.71, 1.87) |  |
| Marital status | 1- Single/Never married (ref) | .. | .. | .. | .. | .. | .. | .. | .. | .. |  |
|  | 2-Married/Common law/cohabitation | 1.48 (**1.20, 1.82**) | 1.71 (**1.29, 2.27**) | 1.31 (0.97, 1.77) | 1.45 (**1.17, 1.79**) | 1.58 (**1.17, 2.12**) | 1.32 (0.97, 1.79) | 1.50 (**1.17, 1.93**) | 1.73 (**1.25, 2.40**) | 1.37 (0.94, 2.01) |  |
|  | 3- Widowed/Separated/Divorced | 1.77 (**1.36, 2.30**) | 1.89 (**1.35, 2.63**) | 1.87 (**1.18, 2.95**) | 1.54 (**1.18, 2.02**) | 1.58 (**1.12, 2.24**) | 1.66 (**1.02, 2.70**) | 1.74 (**1.28, 2.37**) | 1.85 (**1.26, 2.70**) | 1.92 (**1.05, 3.53**) |  |
| Occupation | 1- Employed (ref) | .. | .. | .. | .. | .. | .. | .. | .. | .. |  |
|  | 2- Unemployed | 1.03 (0.88, 1.21) | 1.07 (0.86, 1.32) | 1.05 (0.73, 1.50) | 0.94 (0.80, 1.10) | 1.09 (0.88, 1.36) | 0.89 (0.62, 1.27) | 1.04 (0.86, 1.25) | 1.11 (0.87, 1.40) | 1.06 (0.66, 1.70) |  |
|  | 3- Student | 0.51 (**0.31, 0.86**) | 0.52 (0.26, 1.07) | 0.50 (0.24, 1.01) | 0.41 (**0.25, 0.69**) | 0.41 (**0.20, 0.84**) | 0.41 (**0.20, 0.84**) | 0.80 (0.42, 1.53) | 0.67 (0.28, 1.57) | 1.04 (0.39, 2.75) |  |
|  | 4- Not in the labour force | 1.29 (**1.04, 1.61**) | 1.37 (0.94, 2.00) | 1.36 (**1.03, 1.78**) | 0.98 (0.78, 1.22) | 1.04 (0.70, 1.54) | 0.97 (0.73, 1.28) | 1.23 (0.97, 1.56) | 1.44 (0.96, 2.16) | 1.22 (0.90, 1.66) |  |
| Household size | **-** | 1.01 (0.97, 1.05) | 1.02 (0.97, 1.08) | 1.01 (0.95, 1.06) | 1.00 (0.96, 1.04) | 1.00 (0.95, 1.05) | 1.01 (0.96, 1.06) | 1.02 (0.98, 1.06) | 1.04 (0.98, 1.09) | 1.01 (0.95, 1.08) |  |

*OR, Odds ratio*

(CIs in bold are statistically significant)

Sensitivity analysis 1: elevated blood pressure excluded in the index calculation

Sensitivity analysis 2: anyone with missing data on the index metrics (“index complete case analysis”) excluded.

**Figure S2.** Odds ratio plot of the primary analysis and two sensitivity analyses models for the overall and sex-specific models


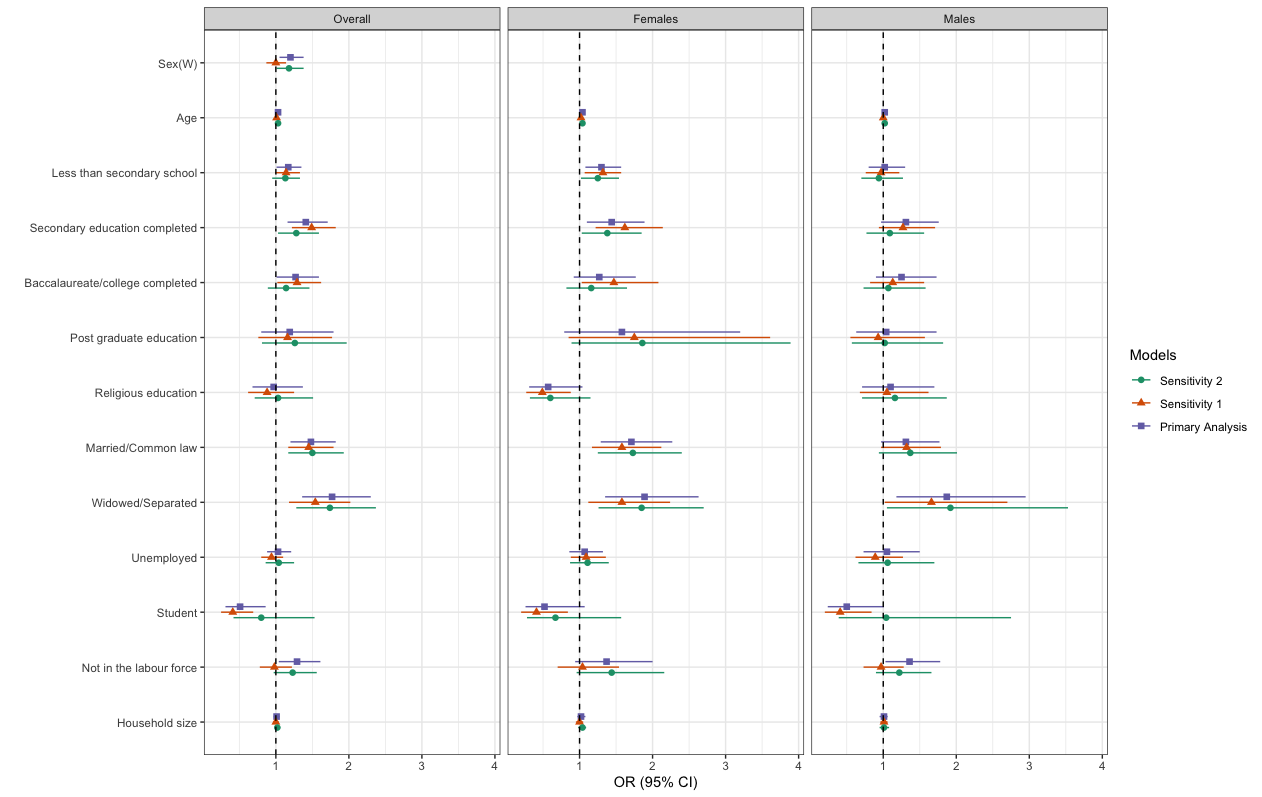


*Reference groups:* sex, men; education, no formal education; marital status, single; occupation, employed

Sensitivity analysis 1: elevated blood pressure excluded in the index calculation (index ranges from 0-4); Sensitivity analysis 2: anyone with missing data on the index metrics (“index complete case analysis”) excluded (index ranges from 0-5).

**Table S12.** Prevalence of diabetes in included STEPS survey datasets

| **Group** | **Prevalence**† |
| --- | --- |
| Overall | (4965/112850)*100= 4.4 |
| In men | (2153/ 46520)*100 = 4.6 |
| In women | (2812/66330)*100 = 4.2 |

† Prevalence of diabetes of non-pregnant adults (18-69 years) is calculated for the STEPS datasets included in the study. People with self-reported prior CVD diagnosis are included in the calculation.
